# Functioning assessment short test (FAST): validity and reliability in adults with Autism Spectrum Disorder

**DOI:** 10.1186/s12888-021-03330-y

**Published:** 2021-06-29

**Authors:** Laura Gisbert-Gustemps, Jorge Lugo-Marín, Imanol Setien Ramos, Gemma Español Martín, Eduard Vieta, C. Mar Bonnín, Josep Antoni Ramos Quiroga

**Affiliations:** 1 Department of Psychiatry, Universitari Vall d’Hebron Hospital, Barcelona, Catalonia Spain; 2 Group of Psychiatry, Mental Health and Addictions, Vall d’Hebron Research Institute (VHIR), Barcelona, Catalonia Spain; 3 Department of Psychiatry and Forensic Medicine, Universitat Autònoma de Barcelona, Barcelona, Spain; 4 Biomedical Network Research Centre on Mental Health (CIBERSAM), Madrid, Spain; 5 Bipolar and Depressive Disorders Unit, Hospital Clinic, Institute of Neurosciences, University of Barcelona, IDIBAPS, CIBERSAM, Barcelona, Spain

**Keywords:** Autism Spectrum quotient, Adult, Functioning, Validity, Reliability, Factor analysis

## Abstract

**Background:**

The assessment of functional impairment is crucial both for the diagnosis and the therapeutic approach to autism spectrum disorder (ASD). The purpose of the present study was to evaluate whether the FAST is a reliable and valid tool to assess functional impairment in adults with Level 1 ASD and to study the differences in psychosocial functioning between younger and older adults with ASD.

**Methods:**

A case–control study was carried out in a sample of 150 participants, 71 adults with Level 1 ASD, and 79 adults without psychiatric history records.

**Results:**

Results showed good psychometric properties in terms of validity and reliability. Cronbach’s alpha for the total scale was .91 and the area under the curve was .98. The study also showed that adults with ASD present different profiles of functional impairment depending on their age: while younger patients present greater impairment in autonomy, older patients show more difficulties in interpersonal relationships.

**Conclusions:**

Our results support the use of the FAST in the evaluation of adaptive functioning in adults with Level 1 ASD.

## Background

Autism spectrum disorder (ASD) is a neurodevelopmental condition with a prevalence of up to 1 out of 54 children and it is characterized by early onset of social-communication difficulties and repetitive or stereotypical behaviors [1]. According to the Diagnostic and Statistical Manual of Mental Disorders, Fifth Edition Disorders (DSM-5) criteria, these symptoms cause “clinically significant impairment in social, occupational, and other fields of current functioning” [2].

The severity of ASD can vary greatly and is based on the degree to which social communication, insistence on the sameness of activities and surroundings, and repetitive patterns of behavior affect the daily functioning of the individual [2]. The term “spectrum” refers to the wide range of symptoms, skills, and levels of disability in functioning that can occur in people with ASD. In fact, adult outcomes are diverse from individuals who remain non-verbal to those able to work and live independently [3].

One of the main therapeutic objectives for individuals with ASD is autonomy in adulthood [4]. Thus, a useful and easy-to-use tool for the assessment of the subject’s functional state is urgently needed to guide the therapeutic effort to obtain the best results.

The assessment of functional impairment, including different areas of a person’s life, such as education, family, social life, working life, leisure and free time [5], is crucial both for the diagnosis and the therapeutic approach to ASD. ASD has been widely studied in children, but the quantity and quality of adult research is scarce [6, 7]. Valid, reliable, and sensitive outcome measures, which are fundamental for the development of an evidence-based for clinical effectiveness, are also lacking [8]. To date, the little existing evidence points to a poor psychosocial outcome for adults with ASD, even in the less severe group [4].

Also, the different stages of adulthood have been studied in relation to the trajectory of people with ASD in different areas of adaptive functioning [9, 10]. However, there have been conducted very few studies that have directly compared the different stages of adult life in the ASD population. Lever et al. [11] studied the prevalence of psychiatric disorders in young, middle and older adults, finding a higher level of psychopathology in young and middle-aged adults when compared to older adults. These differences throughout adult life can be reflected in different levels of functionality, being able to establish different trajectory profiles according to the adult stage. Regarding levels of functionality adaptative behavior measures, particularly daily living skills, have been the variables most closely correlated with outcome in people with ASD [12] and evidence supports that are not fully accounted for by differences in cognitive ability [13]. The curvilinear association between full-scale intelligence quotient (IQ) and adaptive behavior found in the study by Chatham et al. [14] suggests that IQ does not fully explain adaptive behavior difficulties among individuals with ASD, particularly at high IQs where the association between IQ and adaptive behavior is attenuated.

To assess functioning in ASD population, the Vineland Adaptive Behavior Scales, Second Edition (VABS-II) [15] and the Adaptive Behavior Assessment System, Second Edition (ABAS-II) [16] are widely used. The VABS-II is the leading instrument for supporting the diagnosis of intellectual and developmental disabilities and the ABAS-II is a checklist of a broad range of skill areas related to development, behavior, and cognitive abilities. Although they are extensively used, they present some disadvantages, such as their length, making them difficult to apply routinely in a medical setting; moreover, they might not be reliable to assess functioning in people with ASD who do not present intellectual disability. Other measures used to evaluate functionality like The World Health Organization Disability Assessment Schedule II (WHO-DAS II) [17], the SF-36 Health Survey (SF-36) [18], the Global Assessment of Functioning Scale [19] or the Independent Living Scales [20], are not appropriate either due to their length, missing areas of evaluation in a person’s life and not having validation in ASD population. Finally, in ASD and other neurodevelopmental disorders such as ADHD [21, 22] instruments like the Sheehan’s Disability Scale (SDS) [23] are being used because of its easy application despite not having sufficient psychometric properties.

According to this, efforts are needed to adapt the existing instruments and/or develop and evaluate new ones for assessing specific or related outcomes in adult individuals with ASD. Low cost, valid, and not time-consuming measures are needed to facilitate research and monitoring of patients outcome. The Functioning Assessment Short Test (FAST) [24] was created in order to evaluate functional impairment in patients with mental health difficulties. It is a short (6 min to apply) and simple interview-administered instrument which evaluates different domains of functioning regarding the last 15 days before assessment. The higher the score greater difficulties functioning and greater severity [24].

Results of previous studies using the FAST showed optimal validity and reliability properties in patients suffering from different mental health conditions. For instance, in patients with bipolar disorder, high internal consistency for the overall scale (Cronbach’s alpha of .909) as well as for all its domains have been found [24] . In another study with patients with first psychotic episodes [25], an internal consistency level of .88 at baseline, .89 six months later, and .94 one year later have been reported. Recently different severity gradations in first-episode of non-affective psychosis patients have been stablished using this test [26]. The FAST also showed good psychometric properties and could detect functional differences between patients with a diagnosis of schizophrenia and healthy subjects [27]. Finally, Rotger et al. [5] obtained good psychometric properties, in terms of reliability and validity, in the measure of the functional level of adults with ADHD.

Hence, the purpose of the present study is to evaluate whether the FAST could be a reliable and valid tool to assess the functional outcome in adults with ASD in an outpatient clinical setting and to study the differences in psychosocial functioning between younger and older adults with ASD.

## Method

This is an observational case-control study. The study took place in two hospitals from the mental health network of Catalonian Health Institute: Vall d’Hebron Hospital and Barcelona Clinic Hospital. The time window was set in one year and comprised from January 2019 to January 2020.

### Participants

A sample of participants with ASD was recruited from the monitored population within the Comprehensive Care Program for Autism Spectrum Disorder (PAITEA), from the Vall d’Hebron Hospital’s Psychiatry Department. All participants were diagnosed using the Autism Diagnostic Observational Schedule-2 [28] by trained clinicians (L.GG and J.L.M). Inclusion criteria included: 1) being aged between 18 and 65 years; 2) diagnosis of ASD according to DSM-5 diagnostic criteria [2]. Respondents’ language and cognitive barriers and ASD levels 2 and 3 were considered as exclusion criteria [2]. The nonclinical control group (NC) was recruited at the Barcelona Clinic Hospital’s Bipolar and Depressive Disorders Unit [29]. NC inclusion criteria were (1) age between 18 and 65 years, (2) no current or past psychiatric history as collected from medical records, and (3) non-reported history of family psychiatric disorder.

### Assessment

After informing the participants and obtaining their written consent, the investigator recorded demographic (age and gender) and clinical variables (psychiatric diagnosis history, if any) and administered the FAST scale [24]. The FAST is an interviewer-administered instrument, designed to be used by a trained clinician. The evaluation time-window refers to the last two weeks. The areas evaluated with the FAST are six, including: 1) Autonomy, related to the ability of living on their own, taking care of themselves (physical appearance, hygiene …); 2) Occupational functioning, capacity to get and keep a paid job and being efficient at it, working in the field in which the patient was educated, and earning according to the level of his/her position; 3) Cognitive functioning, being able to concentrate while reading or watching a movie, solve problems, remember simple data and learn new information; 4) Financial issues, being capable to manage their own finances; 5) Interpersonal relationships, refers to the capacity of keeping friendships, getting well with family, involvement in social activities, sexual relations, and being able to express and defend own interests; 6) Leisure Time, capacity to exercise and enjoy a hobby. Items (24 in total) are rated on a 4-point scale, ranging from 0 (no difficulty) to 3 (severe difficulty). All scores are added to obtain a global punctuation for which high scores indicate poorer functioning [24].

Additionally, the Sheehan’s Disability Scale (SDS) [23] was also administered. It is a three-item instrument widely used in clinical settings, which measures the severity of disability in three inter-related domains: work, family life/home responsibilities, and social/leisure activities. The patient rates the extent to which responsibilities are impaired by his or her symptoms on a 10-point visual analog scale. Clinicians are recommended to pay special attention to patients who score 5 or higher on any of the three scales because such high scores are associated with significant functional impairment. The SDS has shown good validity and reliability in Spanish clinical population [30].

### Statistical analyses

To study the normality of the FAST variables, Shapiro–Wilk coefficient was applied. The results showed significant deviations from normality, precluding the use of parametric tests. The reliability coefficient (Cronbach’s alpha) was used to examine the internal consistency of the FAST items in each domain and the full scale. Cronbach’s alpha measures were considered minimally acceptable if α = .65, acceptable if α = .7, and optimal if α = .8 [31]. Spearman’s correlation coefficient was calculated to examine the correlations between the FAST and the SDS scores. To explore intergroup differences between ASD versus controls and between younger ASD adults (18–25 years) versus older ASD adults (= > 26 years), nonparametric tests (U Mann-Whitney test) for each FAST domain were conducted. A confirmatory factor analysis (CFA) with the original six-factor structure of the FAST was studied. When evaluating the fit of the CFA and stability models to the data, the root mean square error of approximation (RMSEA) and comparative fit index (CFI) were used. RMSEA values below .06 indicate a good fit to the data and CFI values close to .95 are acceptable [32]. The optimal cut-off point of the measure was analyzed by the area under the ROC curve (AUC). The AUC values were considered minimally acceptable when lower than .7, acceptable when between .7 and .8, optimal when between .8 and .9, and excellent when above .9 [33]. Data analyses were carried out with the statistical package IBM SPSS 19.0 and AMOS 26.0 for Windows. The alpha level was set at *p* < .05.

## Results

The total sample consisted of 150 participants: 71 adults with a diagnosis of ASD level 1 (43 male, 28 female), and 79 adults without psychiatric history records (51 female, 28 male). The mean age in the ASD group was 30.03 years (*SD* = 12, range 18–61), and 39.54 years (*SD* = 11, range 19–55) in the healthy control group (*Z* = − 5.02, *p* < 0.001). The estimated time to complete the FAST was approximately 8 min.

### Internal consistency

Cronbach’s alpha showed excellent value for the FAST total score (α = .97) in the total sample of participants, and also for the ASD group (α = .91), whereas acceptable values were found for the NC group (α = .76). Table 1 shows internal consistency results for each FAST subscale.

**Table 1:** Internal consistency (Cronbach’s alpha) for all FAST subscales and Total score

	Total sample (*n* = 150)	ASD (*n* = 71)	NC (*n* = 79)
**FAST Total score (24 items)**	0.97	0.91	0.76
Autonomy (4 items)	0.92	0.85	0.02
Occupational functioning (5 items)	0.91	0.80	0.91
Cognitive functioning (5 items)	0.90	0.82	0.42
Financial issues (2 items)	0.95	0.93	0.88
Interpersonal relationships (6 items)	0.94	0.82	0.54
Leisure (2 items)	0.56	0.48	0.18

### Convergent validity

Spearman’s correlations analyses were conducted to study convergence between the FAST total score with the SDS scores. Results showed significant small to moderate correlations with each of three SDS domains: Work (*r* = .54, *p <* .001), Social Life (*r* = .27, *p* = .02), and Family Life (*r* = .31, *p =* .01). FAST Occupational Functioning also showed a moderate correlation with SDS Work domain (*r* = .61, *p <* .001); FAST Interpersonal Relationships correlated with the SDS Social domain (*r* = .46, *p* < .001); and the FAST Family Life item (Item 20) correlated with the SDS Family Life domain (r = .68; *p <* .001).

### Intergroup mean differences

Table 2 shows the results of the intergroup mean differences analysis. The ASD group showed significantly higher scores in the FAST total score (*Z* = − 10.23, *p* < 0.001), and in all the subscales when compared to NC participants. When compared by age, younger ASD adults showed significantly lower scores in the FAST Autonomy subscale (*Z* = − 2.31, *p* = .02), whereas older ASD adults showed significantly lower scores in the FAST Interpersonal Relationships subscale (*Z* = − 2.27, *p* = .02). No significant differences in the FAST total score were found between age groups (Table 3).

**Table 2:** Intergroup mean differences (sd) between ASD and NC participants

	ASD (*n* = 71)	NC (*n* = 79)	U Mann-Whitney (Z)
**FAST Total score**	40 (13.55)	4.24 (4.63)	−10.23*
Autonomy	6.08 (3.4)	0.27 (0.63)	−10.27*
Occupational functioning	8.59 (3.84)	0.85 (2.56)	−9.57*
Cognitive functioning	7.62 (3.58)	1.11 (1.27)	−9.75*
Financial issues	2.83 (2.14)	0.2 (.81)	−8.5*
Interpersonal relationships	11.77 (4.4)	1.11 (1.58)	−10.25*
Leisure	3.08 (1.51)	0.7 (1.1)	−8.35*

*p < .05

**Table 3:** Intergroup mean differences (sd) between ASD younger vs. older adults

	ASD younger adults (*n* = 40)	ASD older adults (*n* = 31)	U Mann-Whitney (Z)
**FAST Total score**	39.33 (12.12)	40.84 (15.36)	−.87
Autonomy	6.93 (3.21)	5 (3.39)	−2.31*
Occupational functioning	8.3 (3.23)	8.97 (4.54)	−1.22
Cognitive functioning	7.23 (2.90)	8.13 (4.3)	−.84
Financial issues	3.03 (2.06)	2.58 (2.26)	−.90
Interpersonal relationships	10.9 (4.24)	12.9 (4.41)	−2.25*
Leisure	2.95 (1.34)	3.26 (1.71)	−.93

*p < .05

### Confirmatory factor analysis

**Fig. 1:**
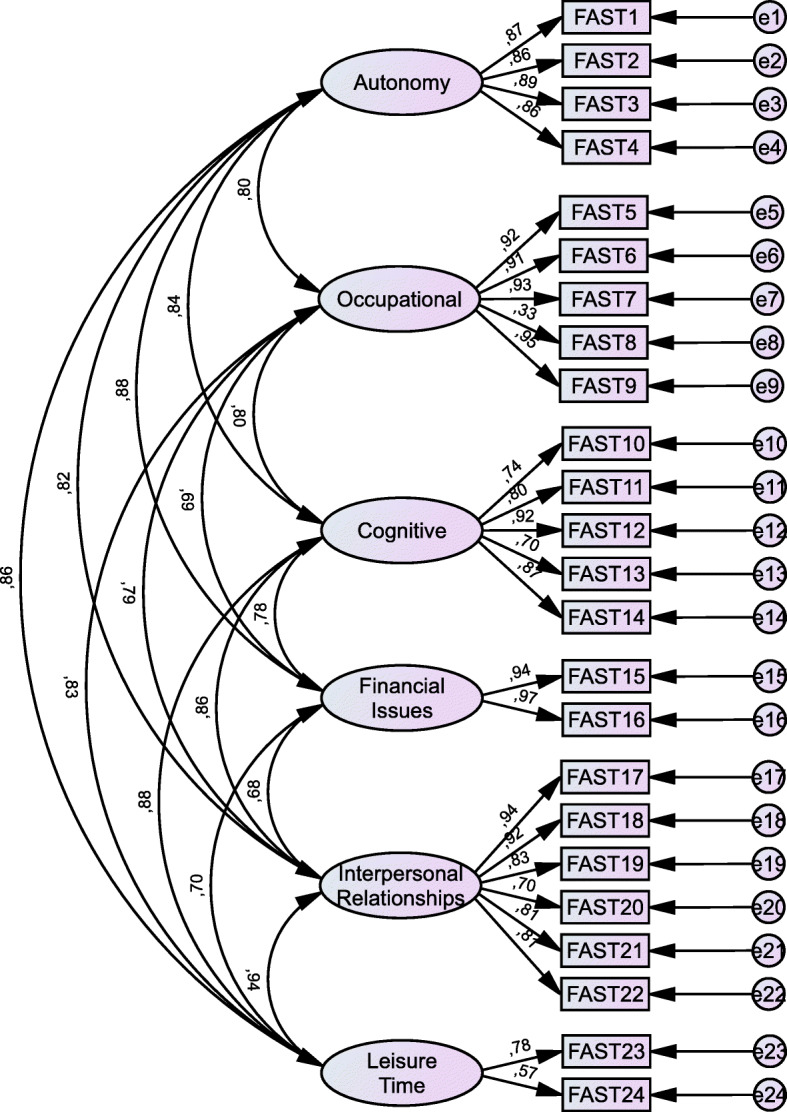
Factor structure of the FAST, including factor correlation and factor loadings (*N* = 150; all subjects)

The study of the internal structure of the FAST determined a poor model fit to the original six-factor structure (Fig. 1). The RMSEA value was .104, (90% CI [.94, .11]; *p* = .00), which is below the acceptable threshold, whereas the CFI value was .90, indicating poor model fit.

### ROC curve

**Fig. 2:**
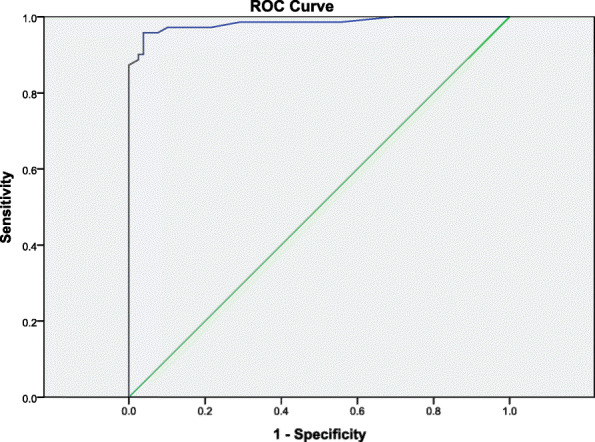
Receiver operating characteristic (ROC) curves illustrating the ability of the total FAST scale to identify any ASD cases at alternative cut-oFf points. *N* = 150 (ASD vs NC)

The AUC was .98, 95% CI [0.96, 1] which, being close to 1, indicates excellent discriminant capacity. A total FAST score above 12 reached best sensitivity (96%) and specificity (94%) scores. Positive and negative predictive values were .96 and .94, respectively. Figure 2 shows the ROC curve for the FAST total score comparing the ASD and NC groups.

## Discussion

The main goal of this study was to analyze the psychometric properties of the FAST scale for the first time in ASD population using a sample of patients with ASD and non-clinical individuals. Results showed good psychometric properties in terms of validity and reliability. Cronbach’s alpha for the total scale was .91 and the area under the curve was .98. Moreover the ASD group showed significantly higher scores in the FAST total score (*Z* = − 10.23, *p* < 0.001), and in all the subscales when compared to NC participants.

A good functional outcome of people with autism is the main objective for professionals, families, and the individuals themselves, but there is no agreement about the instrument to measure it in adults with ASD [34]. At present, there are some limitations to evaluate functioning in autistic people due to methodological issues, particularly related to the wide heterogeneity in the cohorts studied and the variability in the measures used, which have led to inconsistent and sometimes contradictory research findings [4]. Thus more research is needed in this field.

Regarding the psychometric properties of the FAST scale, promising results were found in terms of validity and reliability, very similar to those obtained in bipolar disorder [24], schizophrenia [25], first psychotic episodes [27], and ADHD [5]. Thus our results support the use of the FAST in the evaluation of adaptive functioning in adults with ASD level 1. Moreover, the FAST can be very useful in clinical settings because it is short and very easy to apply. The instrument has been translated to English and validated in different languages, including French [35], Portuguese [36], and Italian [37], among others.

The analysis of the FAST’s internal consistency with Cronbach’s alpha showed good consistency between the different responses and, therefore, between the scale’s items. An excellent alpha index of .91 was found for the ASD group, while an acceptable value, .76, was found for the NC group. This may be because the NC is more heterogeneous than the ASD group, thus providing different functioning profiles and, therefore, a lower consistency between items.

Concerning concurrent validity, modest positive correlations between the FAST total score and scores in each of the three SDS domains were observed. Similar results were obtained in a previous validation of the FAST for ADHD population [5], where the SDS was also used to examine convergent validity. In both cases, the modest correlation obtained could be related to the low similarity between the two measures in the number of items (the SDS with only 3 items and the FAST with 24). In any case, when directly comparing specific social- and family-related items with SDS subareas, higher convergence values were found.

Furthermore, we analyzed the scale’s discriminant capacity between patients and controls using the diagnostic performance or ROC curve. The AUC was .98 which, being very close to 1, indicates an excellent discriminant capacity. These results are in line with those obtained in previous studies regarding bipolar [24] and ADHD populations [5]. The study of the scale’s discriminant capacity also indicates that a score above 12 achieves the best balance between sensitivity (96%) and specificity (94%). Factor analysis showed poor model fit to the six-factor model proposed in the original validation [24]. This may be due to a small sample size. All items showed strong correlations with high factor loadings, except for Items 8 (*Charge according to your position*) and 24 (*Having a hobby*). Further studies should reconsider the original six-factor model when using the FAST in ASD population.

In accordance with previous studies [4, 34], our results indicate poorer functional outcomes for adult people with ASD compared to NC. Most studies of adults with ASD suggest that prognosis, as assessed by objective measures of social outcome (e.g., independence, employment, social relationships), is poor [4]. Considering the age group differences, the younger people showed more difficulties in the autonomy domain. This may be explained by the fact that the sample of younger adults still lived with their parents, compromising their possibilities of developing the self-care necessary skills, as their relatives compensate for their difficulties, which leads to these younger ASD adults’ low motivation to perform these tasks by themselves. On the other hand, older ASD adults show poorer interpersonal relationships, which might be due to constant pressure to “fit in” with the demands of a society that fails to understand their needs or difficulties [38]. Their inability to meet these demands may lead to stress and anxiety and progressive isolation [39]. The older they get, the more social contacts center around special interests and skills, rather than involving close, spontaneous friendships [40].

### Limitations

The findings of this study should be interpreted with caution, considering some limitations that have been identified. First, given that patients and controls were not matched by gender, the number of males in the ASD group is much higher than in the NC group. This is due to a higher prevalence of ASD in male population [41]. Further studies should address gender differences in the adaptive functioning of adults with ASD levels. Also, in relation to age, the older ASD group comprised a wide range of ages (26–65 years). Considering the great variability that can occur throughout the different stages of adult life (middle adult vs. older adult), future studies should explore these differences with greater accuracy. Finally, the FAST might not be appropriate for ASD patients of Levels 2 and 3 due to the content of the items examined. Moreover, differences in cognitive domains (e.g., executive function), as well as adaptive functioning in our sample could not be reported as many of the participants lacked this information. Future research could be conducted in order to adapt some items regarding these variables and thus making the FAST more suitable for the entire ASD spectrum. Among others, items like support needs, communication skills, and cognitive and behavioral difficulties could be taken into account.

## Conclusions

Despite the limitations, these preliminary results point out that the FAST scale presents adequate psychometric properties in terms of validity and reliability, suggesting that it could be an adequate tool to measure of the functioning in adults with ASD level 1. It shows a strong discriminant capacity between ASD and nonclinical subjects. Due to its characteristics, the scale is a feasible measure in healthcare settings, at least to assess patients with ASD level 1. The study also showed that adults with ASD present different profiles of functional impairment depending on their age: while younger patients present greater impairment in autonomy, older patients show more difficulties in interpersonal relationships.

## Data Availability

The datasets used and analysed during the current study are available from the corresponding author on reasonable request.
